# Development and Characterization of Reinforced Flexible Packaging Based on Amazonian Cassava Starch Through Flat Sheet Extrusion

**DOI:** 10.3390/polym18060675

**Published:** 2026-03-11

**Authors:** Johanna Garavito, Sofía Castellanos-González, Clara P. Peña-Venegas, Diego A. Castellanos

**Affiliations:** 1 Food Packaging and Shelf Life Laboratory, Instituto de Ciencia y Tecnología de Alimentos, Universidad Nacional de Colombia, Carrera 30 Número 45-03, Bogotá 111321, Colombia; ngaravitoj@unal.edu.co (J.G.); socastellanosgo@unal.edu.co (S.C.-G.); 2 Instituto Amazónico de Investigaciones Científicas–SINCHI, Avenida Vásquez Cobo, Calle 15/16, Leticia 910001, Colombia; cpena@sinchi.org.co

**Keywords:** thermoplastic starch, pelletization, ground plantain leaf, beeswax, moisture stability

## Abstract

Thermoplastic starch (TPS) can be a sustainable alternative to petrochemical plastics for flexible packaging, especially in rainforests and tropical regions where native starch sources such as cassava are abundant. However, one problem preventing TPS packaging from widespread use is its susceptibility to moisture. This study evaluated TPS formulations based on Amazonian cassava starch reinforced with plantain leaf fibers, beeswax, and low-density polyethylene (LDPE) particles. The plastic compounds were extruded to obtain pellets and then films at 120–130 °C. The resulting films were then cut and heat-sealed to obtain flexible packaging. Different properties of the TPS packages were evaluated, such as mechanical strength, water vapor transmission (WVTR), color, infrared spectrum (FT-IR), and moisture adsorption. The results showed that the formulation with beeswax (2% *w*/*w*), plantain leaves powder (1% *w*/*w*), and LDPE powder (2% *w*/*w*) had a higher tensile strength (5.99 MPa) and moisture barrier (WVTR = 366.6 g m^−2^ d^−1^) compared to the control formulation only with plasticizers (glycerol and water) but without reinforcements (0.48 MPa and 1486.6 g m^−2^ d^−1^, respectively). Films with only beeswax (4% *w*/*w*) and plantain leaves powder (2.5% *w*/*w*) had tensile strength = 5.53 MPa and WVTR = 716.8 g m^−2^ d^−1^, with higher moisture adsorption compared to the samples with LDPE. In both cases, homogeneous and heat-sealable bags were obtained. The reinforced TPS films can be used to reduce the environmental impact generated by single-use packaging applications such as food commercialization.

## 1. Introduction

Using starch as a plastic material for packaging has gained significant attention recently due to the increased concern about the environmental impact of traditional polymers [1,2,3]. Thermoplastic starch (TPS) can become a sustainable and viable alternative to replace petroleum-derived plastics, especially in the production of single-use flexible packaging, mainly considering its high biodegradation rate at ambient conditions and low requirements for home composting [4,5,6]. Replacing low-sustainability petrochemical plastics is imperative for the planet’s environmental balance. In places of high biodiversity, such as the Amazon, the incidence of pollution has become alarming, as the presence of plastics has already been reported in waters [7], alluvial soils [8], fish [9,10], and terrestrial animals such as ants [11] and bats [12]. Furthermore, this contamination has been associated with the proliferation of insects that carry human diseases, suggesting that the problem could be more serious than previously considered.

There are practical problems in replacing traditional materials with more sustainable ones, such as starch. Raw materials are available for significant production [13] and obtaining packages with appropriate functional characteristics. As for the first, in regions such as the Colombian Amazon, some households grow small crops of products such as cassava, from which sufficient surpluses can be generated to produce thermoplastic starch without affecting food security in the region [14,15,16]. Since it comes from small farms in broad geographical areas, different varieties of cassava are used according to the possibilities of each small producer [17]. Likewise, starch isolation is mainly done by hand, which is why there can be much heterogeneity in the raw material obtained. This implies the need for prior characterization of the starch in packaging to determine its composition, amylose/amylopectin ratio, and moisture content [18,19].

Several authors have explored using starch for flexible packaging, such as bags and pouches [20,21]. However, some starch characteristics are unsuitable, especially its low mechanical strength and high sensitivity to moisture [22,23]. For the production of flexible packaging, TPS has a water vapor transmission rate (WVTR) of 1104.0 to 1120.8 g m^−2^ d^−1^ [24], tensile strength of 1–5 MPa and Young’s modulus near 3–22 MPa [25,26], and moisture absorption ranges from 0.035 to 0.091 g g^−1^, depending on the starch source and the type of plasticizer considering that the latter increases active site availability by exposing hydrophilic hydroxyl groups [27,28]. Additionally, relative humidity plays a key role in water uptake and the film’s structural stability [27], which limits its usefulness in the packaging of food and high-moisture products, or under high humidity and temperature conditions [29,30].

One option for improving the characteristics of thermoplastic starch in the manufacture of flexible packaging is its reinforcement and/or compounding with other materials [31]. Currently, many commercial flexible packages that include starch do so using formulations with high amounts of different polymers such as poly (lactic acid) (PLA), polybutylene adipate terephthalate (PBAT), and low-density polyethylene (LDPE) [32,33,34]. Regarding reinforcement materials, using different fillers for plastic compounding, such as cellulose fibers, clays, metal oxides, waxes, and agro-industrial waste, has been explored [35,36]. These materials are included at micro- and nanoscopic scales to obtain a highly homogeneous dispersion using appropriate plasticizing, cross-linking, and emulsifying additives [37].

Several studies have been developed to improve the properties of TPS using reinforcing materials. Siriwong et al. [38] investigated using cellulose fibers isolated from sugarcane bagasse as reinforcement in starch films, obtaining a significant improvement in the water vapor barrier due to the formation of an interconnected network between starch and cellulose fibers. Jeencham et al. [39] developed reinforced TPS films reinforced with microcrystalline cellulose, obtaining a tensile strength of 3.21–11.18 MPa and Young’s modulus of 112.12–488.89 MPa, respectively. For their part, Marta et al. [40] utilized chemically modified starch nanocrystals to enhance bioplastics, improving tensile strength, thermal stability, and biodegradability while reducing water vapor permeability. However, excessive concentrations led to aggregation, diminishing overall performance and effectiveness. Jumaidin et al. [41] incorporated heat-treated plantain leaf fibers into thermoplastic cassava starch (TPS), enhancing the biocomposites’ thermal stability, mechanical properties, and sustainability, with optimal performance at 40 wt.% fiber content. Finally, González et al. [42] plasticized starch nanocomposited films reinforced with graphene (G) and graphene oxide (GO), prepared through solvent casting using Salvia extracts as surfactants, exhibited notable enhancements in mechanical properties and electrical conductivity, reaching values up to 9.0 × 10^−4^ S m^−1^ after GO reduction. The studies emphasized the importance of selecting appropriate additives, such as compatibility or functionalization agents, and ensuring thorough mixing of the components to achieve a homogeneous plastic matrix with well-dispersed reinforcing elements, resulting in superior mechanical strength and barrier properties following casting or extrusion processes.

Considering the above, in this study, cassava starch from Amazonian regions was used to obtain flexible packaging through flat sheet extrusion, and reinforced with plantain leaf microfibers, beeswax, and LDPE microparticles, which were evaluated as a transitional reinforcement strategy to enhance mechanical stability and moisture resistance under the high humidity and temperature conditions typical of the Amazon. This approach aims to establish functional performance benchmarks for starch-based packaging systems in remote tropical regions, while substantially reducing the content of non-biodegradable material and supporting future substitution by fully biodegradable alternatives.

## 2. Materials and Methods

### 2.1. Starch

**Figure 1:**
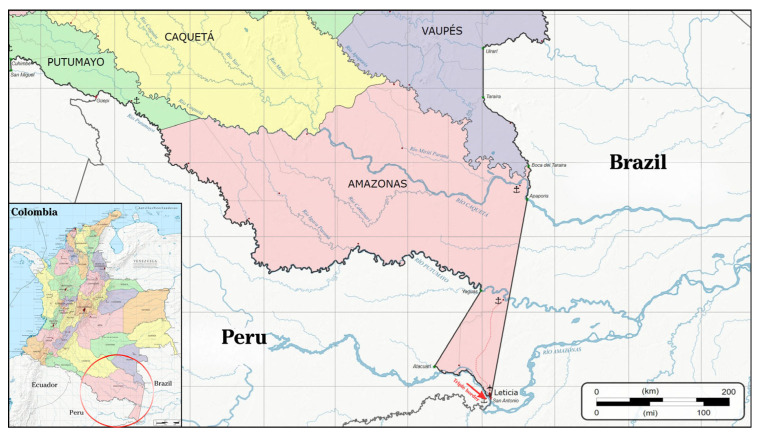
Location of Amazonas Department, and the city of Leticia in Colombia. Adapted from [43] under the Creative Commons Attribution (CC-BY-SA-3.0).

Raw cassava starch was collected from 10 indigenous communities near the so-called triple border between Brazil, Colombia, and Peru (Figure 1), and close to Leticia (Amazonas Department, Colombia, 25/38 °C and 76/90% RH). In this region, small-scale Indigenous producers cultivate cassava in small plots of land known as ‘Chagras.’ Samples of several polyclonal starch varieties from native species of the area were collected and mixed into a single final material. For this study, it was decided to proceed in this way, considering the low representativeness of each variety for possible future scaling-up processing. After obtaining the raw mixture, bromatological measurements were conducted as indicated below.

**Figure 2:**
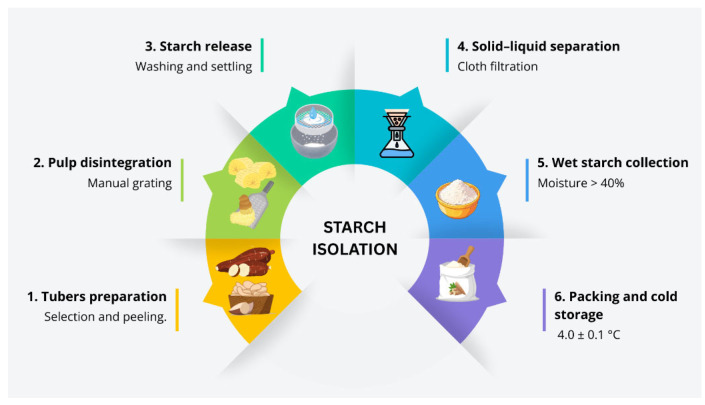
Simplified schematic representation of the starch isolation process from cassava.

Starch samples were isolated by hand (Figure 2), initially removing the tuber’s peel and then grating the pulp. The grated pulp was then washed and left to rest, allowing the starch to settle, which was then filtered through a sheet of fine cloth to drain off excess moisture. The wet starch mass with a moisture content > 40% was then packaged in low-density polyethylene (LDPE) pouches (0.05 mm thick) and sent within 24–48 h to the facilities of the Food Packaging and Shelf-Life Laboratory of the National University of Colombia (Bogotá, Colombia), where it was kept at 4.0 ± 0.1 °C until conditioned.

### 2.2. Plasticizers and Additives

For the extruded film production tests, glycerol (Necardis S.A.S., Bogotá, Colombia; 99.8% purity) was used as a plasticizer, sorbitan monooleate (Span) 80 supplied by Sigma-Aldrich (Sigma-Aldrich Canada Ltd., Oakville, Canada; 99.5% purity) was used as an emulsifier, and citric acid (Shandong Ensign Industry Co., Ltd., Weifang, China; 99.5% purity) was used as a cross-linking agent.

### 2.3. Reinforcing Compounds

For the inclusion tests with reinforcing components, latex extracted from the *Hevea brasiliensis* trees from the Guaviare region in Colombia, solid beeswax (Adbaquim S.A.S., Bogotá, Colombia), and sunflower oil (La Fabril S.A., Montecristi, Ecuador) were used. Likewise, low-density polyethylene (LDPE) (SABIC, Riyadh, Saudi Arabia; Ref. 1922N0) and plantain (*Musa paradisiaca*) leaves from small local crops near Leticia (Amazonas, Colombia) were also used. The leaves obtained for the study are usually discarded as waste from plantain production.

### 2.4. Conditioning of Raw Materials

Before extrusion testing, starch was dried in a three-tray convection dehydrating oven (Comek, Bogotá, Colombia) at 40 ± 2 °C until obtaining a target moisture content of 10.0 ± 2.0%, defined as the conditioning range required to ensure stable extrusion processing. Moisture content was measured using a Mettler Toledo HR73 halogen moisture analyzer (Mettler-Toledo International Inc., Columbus, OH, USA) (AOAC 925.10-1925 [44]). Once dried, starch was ground in a hammer mill pulverizer (Maquinaria & Soluciones Alimenticias S.A.S, Bogotá, Colombia) and sieved with a Number 40 mesh sieve down to an average particle size of <0.40 mm. Plantain leaves were dried down to a moisture of 7.7 ± 0.1% and then ground to a particle size < 0.15 mm. The LDPE was pulverized from blocks to a particle size of <0.40 mm. The latex was hydrolyzed by adjusting the pH to an optimal value of 3, adding citric acid, and incorporating hydrogen peroxide (PanReac Química SLU, Barcelona, Spain) at 5% (*w*/*w*) by continuous dripping while maintaining constant agitation for 24 h at 40 ± 2 °C.

### 2.5. Characterization of Amazonian Starch

Physicochemical analyses were performed on the mixture of samples of polyclonal cassava starches collected from the local Amazon communities. Moisture content was determined using a Precisa EM 120-HR Moisture Analyzer (Precisa Gravimetrics AG, Moosmattstrasse, Dietikon, Switzerland) and following the AOAC method 945.15 [45]. Ash content was determined using a Terrigeno Model D8 calciner (Compañia Terrigeno S.A.S., Medellin, Colombia) following the AOAC method 942.05 [46]; ether extract was measured using the AOAC 920.39 method [47], protein content was obtained by the AOAC 920.87 method [48], and fiber and carbohydrates by the AOAC 991.43 method [49]. All the measurements were expressed as mean values ± standard deviation for 10 replicates.

### 2.6. Formulation and Premixing to Produce Films

After performing preliminary tests, five formulations were preselected and prepared, with cassava starch serving as the primary polymeric matrix (between 60.1 and 66.6% *w*/*w*), while varying the inclusion and composition of additives and reinforcing components. Glycerol (23–25% *w*/*w*) and water (6.9–7.4% *w*/*w*) were included as plasticizers, citric acid (1% *w*/*w*) as a crosslinker [50,51], Span 80 (0–0.5%) as an emulsifier to stabilize the emulsion, and sunflower oil, hydrolyzed beeswax latex, powdered plantain leaves and powdered LDPE (0–4% *w*/*w* each) as a moisture barrier and mechanical strength reinforcements.

The formulations shown in Table 1 were pre-selected using a process-oriented feasibility approach rather than a classical factorial experimental design. Preliminary extrusion and film-forming tests were conducted to evaluate the combined effect of additives and reinforcing components on the thermoplastic starch matrix, focusing on: (1) smooth flow through the extruder without blockages; (2) homogeneous pellets without granules or various visible solid phases; (3) reinforcing compounds perfectly mixed in the pellet and film without exudation or partial phase separation; (4) continuity of the film in the flat sheet extrusion without defects such as “fish eyes” formation, breakage or excessive adhesiveness.

#### 2.6.1. Technical Constraints and Selection Criteria

The selection of the final formulations was based on processing constraints observed during the preliminary extrusion test. The starch was conditioned up to a moisture of 10.56 ± 1.01% to avoid excessive instability due to water evaporation in the extruder and excessive gelatinization at higher values [52]. Regarding plasticizer, glycerol concentrations were set at 23–25% (*w*/*w*) to avoid inducing overplasticization with larger levels [27]. The inclusion of reinforcing agents was also limited by melt stability and phase separation. For beeswax homogenization, Span 80 was used because it achieved better dispersion than Tween 80 in the preliminary test. In this case, the Span 80 concentration was set at 0.5% (*w*/*w*), considering the study by Ortega-Toro et al. [53]. Sunflower oil and beeswax were capped at 4% (*w*/*w*) to avoid loss of stability in the dispersion due to the low affinity between these components and the hydrophilic starch matrix and plasticizers (water and glycerol) [54]. Similarly, LDPE was limited to 2% *w*/*w*, and plantain leaf powder was restricted to 2.5% (*w*/*w*) to keep a stable dispersion and avoid the use of additional compatibilizing additives [55].

#### 2.6.2. Component Mixing Process

**Figure 3:**
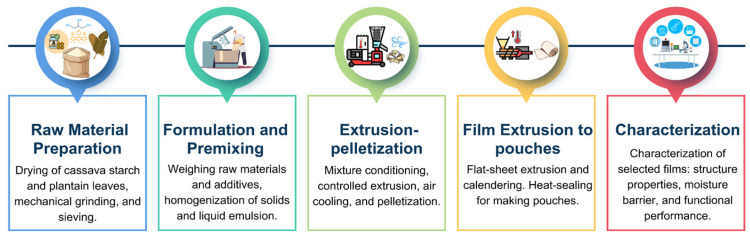
Experimental process for cassava starch-based film production and characterization.

For the component mixing process, the solid components (powdered plantain leaves, starch, and citric acid) were manually pre-mixed and homogenized to ensure uniform distribution. On the other hand, the beeswax was heated at 60 °C until it completely melted, after which the impurities were filtered out at a constant temperature. At the same time, glycerol was heated separately to 60 °C, after which it was mixed with beeswax and Span 80 until fully integrated, forming a stable liquid mixture. This emulsion was then transferred to a new container, where the solid mixture was gradually incorporated into the liquid phase, providing complete integration. For formulation F5, pulverized LDPE was added last and thoroughly homogenized to ensure even dispersion throughout the mixture. The sequential stages of the experimental process, from raw material conditioning to final film characterization, are summarized in Figure 3.

### 2.7. Extrusion–Pelletization

Initially, 1 kg of a mixture of each of the formulations to be evaluated was prepared, besides the control. These were left to rest for 24 h in closed containers at room temperature to facilitate the complete diffusion of the plasticizers throughout the material. The mixtures were fed into a Bimek BK30 extruder (Bimek S.A., Bogotá, Colombia), with a 2-inch diameter screw and an L/D ratio = 25. The extruder consists of 4 heating zones, which were set at 120 °C for the feeding zone, 125 °C at the compression zone, 130 °C in the metering zone, and 135 °C at the die, while the screw speed was set to 60 rpm to ensure adequate plasticization, preventing the mixture from becoming sandy or excessively liquid. These conditions were set after preliminary testing for the fine-tuning of the process and based on other extrusion studies for TPS [56,57,58]. The extruded strands were cooled by a stream of dry air before cutting, allowing adequate solidification for pelletization. Finally, the strands were cut into pellets with approximate dimensions of 3 ± 2 mm in diameter and 5 ± 1 mm in length.

### 2.8. Film Extrusion

Another extruder of the same model and brand as the one used for the strands’ extrusion was used to obtain the films, but equipped with a flat sheet die with an effective output length of 21.5 cm and an opening between lips of 1.04 mm. The pellets obtained for each formulation were fed into the extruder, which was adjusted to a screw speed of 60 rpm and with a temperature profile of 120–125–130 °C from the feeding to the metering zones and 135 °C for the die. At the exit of the extruder, the molten sheet was transferred to a calender equipped with water-cooled rollers, setting the pulling speed at 4 rpm and the opening of the rollers at 0.5 mm to ensure a uniform thickness. The calender’s pulling speed was adjusted to obtain continuous films with thicknesses of 0.2–0.5 mm.

### 2.9. Making of Pouches

The films obtained from each formulation were initially cut to obtain 15 cm × 20 cm pouches from a single rectangular piece. After cutting, the piece was folded in half and heat-sealed in an impulse sealer type PFS-400 (Americas Maquinaria S.A.S., Bogotá, Colombia) at an approximate sealing temperature of 120 °C. The heat-sealing process started with a contact time of 3 s, sealing the lateral ends of the sheets to form a continuous bag. Once the bags of each film formulation were obtained, characterization tests were performed to evaluate their physical and stability properties, thus determining the most favorable formulation obtained for flexible packaging.

### 2.10. Film Characterization

Functional groups of the components present in the films were identified by modulated mid-infrared analysis with a LYZA 7000™ FTIR-spectrophotometer (Anton Paar GmbH, Graz, Austria) at room temperature and measuring absorbance in the region of 4200–500 cm^−1^ with the Diamond attenuated total reflectance (ATR) cell. The measurements were performed with a spectral resolution of 8 cm^−1^, making 48 scans per sample to ensure a high signal-to-noise ratio.

The surface and cross-sectional morphology of selected extruded films was examined by scanning electron microscopy (SEM) using a Dual Beam System Tescan Lyra 3 (Tescan Group, Brno, Czech Republic) operated at an accelerating voltage of 5.0 kV. Before observation, the samples were sputter-coated with a thin gold layer (~10 nm) to ensure adequate electrical conductivity. Cross-sectional images were obtained by carefully cutting the films with a fine blade. The SEM micrographs were used to assess surface continuity, internal structure, and the dispersion of reinforcing components within the thermoplastic starch matrix.

The film’s thickness was determined with a Mitutoyo^®^ Digimatic IP65 digital micrometer (Mitutoyo, Kawasaki, Japan), taking measurements at three different points on each sample and reporting the mean value in mm.

The samples’ color was measured on the surface of the films using a 3nh YS3020 spectrophotometer–colorimeter (Shenzhen 3nh Technology Co., Ltd., Guangzhou, China), reporting the L*, a*, and b* coordinates of the CIELAB color space. A standard illuminant, “Daylight 65,” and a 10° observer were considered. Luminous transmittance (%) was estimated from the reflectance value, for a wavelength of 600 nm, by using the same equipment mentioned before [59].

The water vapor transmission rate (WVTR) and water vapor permeability coefficient, water vapor adsorption, and mechanical strength of the films were determined at 35 ± 2 °C and 75 ± 5% RH, simulating conditions like those expected in the Leticia area (Amazon, Colombia). In each case, the samples for each test were introduced into an ICH110 climatic cabinet (Memmert GmbH + Co.KG, Schwabach, Germany). The climate cabinet was maintained under forced air working conditions at maximum adjustable fan speed capacity to allow homogeneous conditions.

The water vapor permeability coefficient was determined following the ASTM E96/E96M-24 standard [60], based on gravimetric weight loss analysis (Water Method). Initially, distilled water (5 ± 0.05 g) was introduced into a 35 mm diameter aluminum cylindrical cell with a permeation area of 0.00096 m^2^, and the test film was then placed on top of the cell. The weight loss of the evaporated water (permeated through the film) was recorded until a constant loss rate was reached, and with the differentials of lost weight, the calculation of the water vapor transmission rate (WVTR, in g m^−2^ d^−1^) was performed. From this, the permeability coefficient of each material (Q_H2O_) was estimated (in g mm m^−2^ atm^−1^ d^−1^) considering the thickness and permeation area of the film.

The sample’s moisture (water vapor) adsorption was determined by performing a methodology similar to the ASTM D570-22 standard [61]. Samples of 100 mm × 20 mm were used and preconditioned in an oven at 50 °C for 24 h before testing. Moisture adsorption was estimated based on the increase in sample weight relative to its initial weight while exposed to the controlled conditions [59]. The moisture adsorption of the films was measured at 6 and 24 h after being placed in the climatic cabinet.

On the other hand, additional samples were introduced to determine the tensile strength based on the same storage conditions, taking measurements at 0, 6, and 24 h. In this way, the effect of high humidity and temperature conditions was evaluated on the mechanical properties of the films. Tensile strength was determined according to the ASTM D882-18 standard [62]. For this, 100 mm × 20 mm specimens were prepared and placed in a Lonroy LR-C001 tensile machine (Dongguan Lonroy Equipment Co., Ltd., Dongguan, China), where they were stressed to failure at an elongation rate of 10 mm s^−1^. Peak force (N), tensile strength (MPa), elongation at break (%), and Young’s modulus (MPa) were recorded.

Measurements of color, transparency, water vapor permeability, and moisture adsorption and mechanical tensile strength were performed in triplicate (*n* = 3), reporting each case’s mean value and standard deviation. For the mechanical resistance measurements, 5 replicates per sample (*n* = 5) were used.

### 2.11. Statistical Analysis

A General Linear Model (GLM) ANOVA was performed at a 95% confidence level to compare the different formulations according to their property measurements. The statistical analysis was conducted using Minitab^®^ Statistical Software 22.2.1 (State College, PA, USA: Minitab, LLC). The model included treatment, time, and interaction as fixed factors to assess their individual and combined effects on the response variable. Following the ANOVA, Tukey’s HSD test method was applied to determine which means differ significantly from others within each factor level. The multiple comparisons were carried out separately for treatment, time, and the treatment × time interaction to ensure an accurate identification of significant differences.

## 3. Results and Discussion

### 3.1. Starch Characterization

Table 2 presents the physicochemical characterization of polyclonal starches extracted from ten regional farming communities. The table includes key parameters such as moisture content, ash content, protein levels, and carbohydrate composition, providing an overview of the average quality and variability of starches from diverse varieties and local farming practices.

When comparing the physicochemical parameters of the Amazonian cassava starch (Table 2) with the standards established by the FAO technical guides for the production and analysis of this product [63], variations in different compositional characteristics can be observed. The moisture content (44.15 ± 0.27%) significantly exceeds the recommended commercial range for starch (10–13%), due to the local practice of vending high-moisture material for food preparations. This approximation makes the extracted starch highly susceptible to microbial deterioration and fermentation. Similarly, the ash content on a dry basis (0.29%) surpasses the suggested limit (<0.12%). This higher mineral residue can be attributed to soil contamination during harvesting and the lack of a post-harvest cleaning stage in the traditional extraction process used by indigenous communities in the cultivation area. The above corresponds to a higher fiber content (0.13% on a dry basis), which reflects artisanal filtration techniques, which are typically less effective than commercial methods. Regarding protein, a value of 1.21% (on a dry basis) was recorded as shown in Table 2, much higher than usual for cassava starch of 0.4% [64,65]. In this case, variations in protein content could significantly influence the flexibility and stability of starch-based materials. A higher protein content can enhance film elasticity by improving the tensile properties of the starch matrix [66], while lower protein levels indicate greater starch purity and could increase water permeation [67].

Despite the differences with values reported for other cassava starches for the different components mentioned above, Amazonian starch maintains a high carbohydrate content on a dry basis, as shown in Table 1, aligned with the FAO range (92–98%) and evidencing that even rudimentary isolation methods can produce starch of satisfactory purity [63]. This degree of purity is sufficient for obtaining cassava starch films. Criollo-Feijoo et al. [68], for example, prepared active films with starches extracted from cassava bagasse with a purity close to 83%, showing satisfactory results and only some differences in color and elongation compared to industrially extracted cassava starch. In this case, the starch molecules make possible the formation of strong, cohesive films through hydrogen bonding among their chains. Starch’s two main components play distinct roles in determining the mechanical and structural properties of the films; amylose contributes to rigidity and strength, and amylopectin enhances flexibility [5]. Cassava starch is particularly valued for its excellent film-forming capacity, which is attributed to its amylose-to-amylopectin ratio of approximately 17/83% (*w*/*w*), in addition to offering other advantages such as a low gelatinization temperature, high gel stability, and high transparency [69,70].

### 3.2. Conditioning of Starch, Formulation, and Pellet Formation

**Figure 4:**
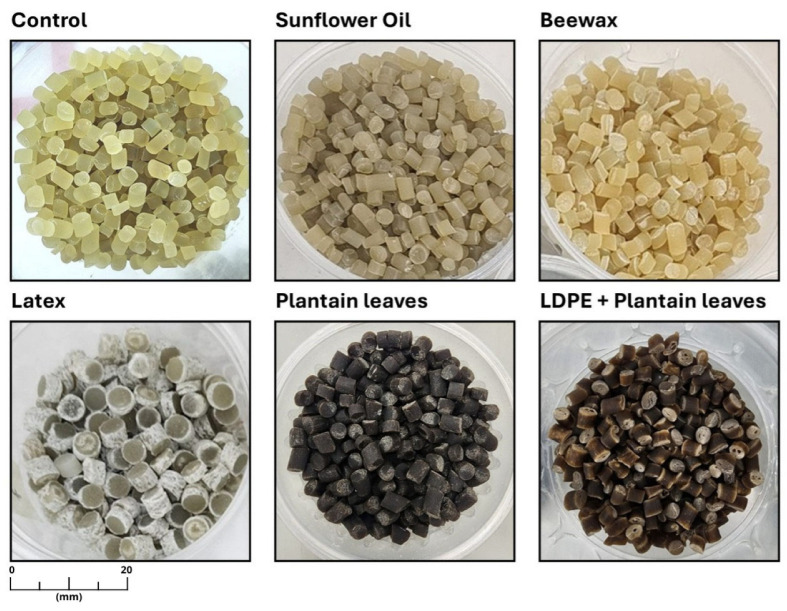
Pellets obtained from the extrusion of different compounded TPS formulations. All images are shown at the same scale; the scale bar represents 20 mm.

Figure 4 shows the pellets obtained for the formulations shown in Table 1 after the preliminary tests. Control samples, composed solely of starch without reinforcements, resulted in crystalline, translucent, and slightly yellowish pellets, with a homogeneous cylindrical shape and smooth surface, indicating good internal cohesion and matrix stability. However, some plasticizers’ migration and rapid moisture absorption were observed, which can affect mechanical stability over time [71]. Formulation F1, containing sunflower oil, exhibited minor irregularities in shape, primarily associated with fluctuations in the filament during extrusion and a noticeably tacky surface reflecting the high adhesiveness induced by this lipophilic agent, consistent with other studies indicating that vegetable oils can hinder processability due to poor compatibility with hydrophilic matrices [72,73]. On the other hand, formulation F2, incorporating beeswax, yielded well-formed, firm, and dimensionally stable pellets, supporting findings that natural waxes enhance moisture resistance and hydrophobicity without compromising material flexibility [74]. F3, which included natural latex, exhibited clear signs of thermal incompatibility even at this early stage: the pellets displayed a whitish, rough outer layer, indicative of material degradation and phase separation, affecting the uniform distribution of latex within the matrix, a phenomenon also reported in systems with non-compatible polymers [75,76]. For F4, which included plantain leaves, the pellets were dark, rough, and visibly homogeneous, reflecting good dispersion of lignocellulosic particles. This physical reinforcement in starch matrices enhances mechanical stability and reduces adhesiveness [77,78]. Finally, formulation F5, which combined LDPE with plantain leaves, yielded similar results to those of F4 in the pelleting stage, except that a less intense brown color was observed; it was more shiny and rigid, with rigid LDPE microparticles that reinforce the polymer structure [79,80]. The extrusion and flat sheet calendering stages played a critical role in determining formulation viability, as minor differences in melt cohesion and thermal stability strongly influenced film continuity and thickness control. These process-dependent effects highlight the importance of evaluating thermoplastic starch formulations under extrusion conditions rather than relying solely on compositional criteria.

### 3.3. Sheet Extrusion and Bag Formation

The obtained pellets were processed by flat sheet extrusion, revealing significant variations in material behavior during calendering, winding, and heat sealing. The control formulation produced a slightly translucent, initially flexible film. However, it proved very fragile during calendering and winding, and its properties degraded rapidly under environmental conditions. Over time, high moisture loss was observed, making the samples increasingly stiff and brittle. Additionally, although the control film was heat-sealable at 170 °C for 3 s, subsequent exposure to ambient moisture weakened the seals, leading to layer separation. These observations underscore the inherent challenges of plasticizer migration and sensitivity to environmental moisture in pure starch matrices, which negatively impact the mechanical strength and stability of the resulting films [81].

A detailed analysis of the reinforced formulations revealed specific processing limitations that led to the exclusion of F1, F2, and F3. The formulation containing vegetable oil (F1) exhibited good continuity and initial elasticity but showed excessive adhesiveness and sheet fusion during winding. This prevented heat-sealing tests because it was impossible to unwind the rolls or achieve proper cuts, which is consistent with plasticizer migration and the inherent adhesive properties of fatty agents in hydrophilic matrices [82]. In contrast, the formulation with beeswax (F2) eliminated adhesiveness and significantly improved stability during calendering and winding, highlighting the hydrophobic and stabilizing properties of natural waxes in biopolymer matrices as reported by [74,83]. However, this formulation was discarded due to insufficient mechanical resistance during the bag formation. Finally, the latex-based formulation (F3) exhibited high incompatibility between components and evident fragility, with non-homogeneous areas and a rough surface texture. The seals obtained with this formulation were weak and easily disintegrated during bag forming, a behavior attributed to thermal degradation of the latex and changes in its molecular structure at high temperatures [84].

**Figure 5:**
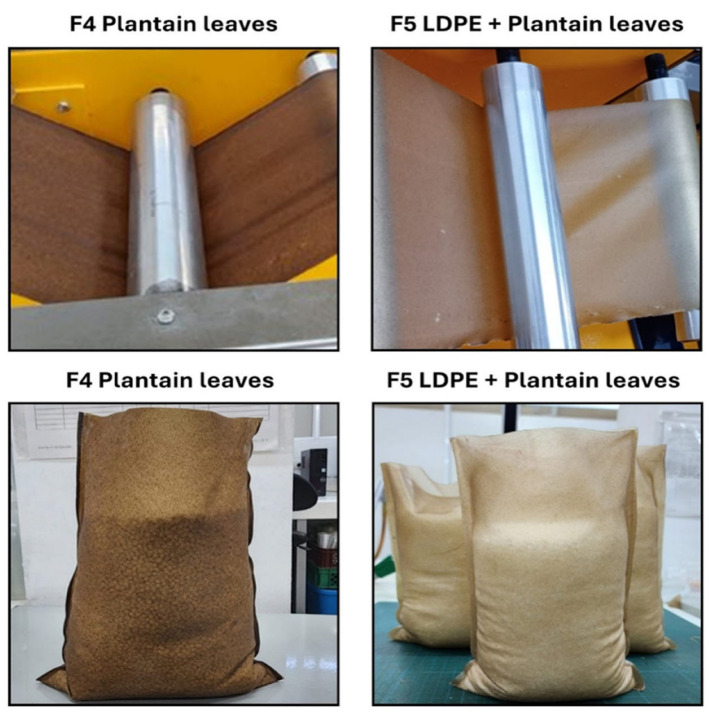
Calendering process for film obtention and heat-sealed bags of TPS formulations with ground plantain leaves (F4) and LDPE powder + ground plantain leaves (F5).

In contrast to the discarded formulations, Formulation F4, which incorporates beeswax and ground plantain leaves, showed good calendering stability and heat-sealing performance. The resulting bag prototypes achieved consistent cuts and side seals, though minor surface imperfections were observed due to agglomeration of plantain leaf particles. Meanwhile, formulation F5, which combined the ground plantain leaves with the LDPE powder, produced films with superior plastic properties, better homogeneity, and no adhesion issues during winding (Figure 5). Regarding heat sealing, strong, durable side seals were achieved for both F4 and F5 films, maintaining structural integrity during handling without delamination or weakening. For these optimized formulations, the sealing temperature was maintained at 170 °C, with sealing times adjusted to 5–6 s to ensure robust bonding.

After the bags were formed and heat-sealed, a small seal capacity test was performed, introducing approximately 1 kg of starch pellets over 24 h to determine if the seals held properly. For formulations F4 and F5, the seals were maintained, preserving the bag structure with the contained material, as shown in Figure 5. F5 heat-sealed bags retained their structure and seals better after one day with contained material. In the case of the control formulation, there was rapid weakening and separation of the seals due to moisture absorption as previously mentioned. The findings achieved align with recent studies indicating that small amounts of synthetic polymers like LDPE can enhance biopolymers’ processability and mechanical properties without compromising their environmental integrity [37,85,86].

### 3.4. Film Characteristics

Considering the best characteristics of homogeneity and stability during the process of extrusion (pellets and flat sheet formation) of formulations F4 and F5, the characterization tests were carried out for the films obtained from these two formulations, in addition to the control formulation as a comparison criterion. Formulations F1 and F3 were discharged since those formulations exhibited significant defects such as fragility, adhesiveness, or thermal incompatibility as described above. Formulation F2 was also discarded because it resulted in a very flexible (too stretched in the calender) and adhesive sheet.

#### 3.4.1. Infrared Spectroscopy (IR) of the Films

**Figure 6:**
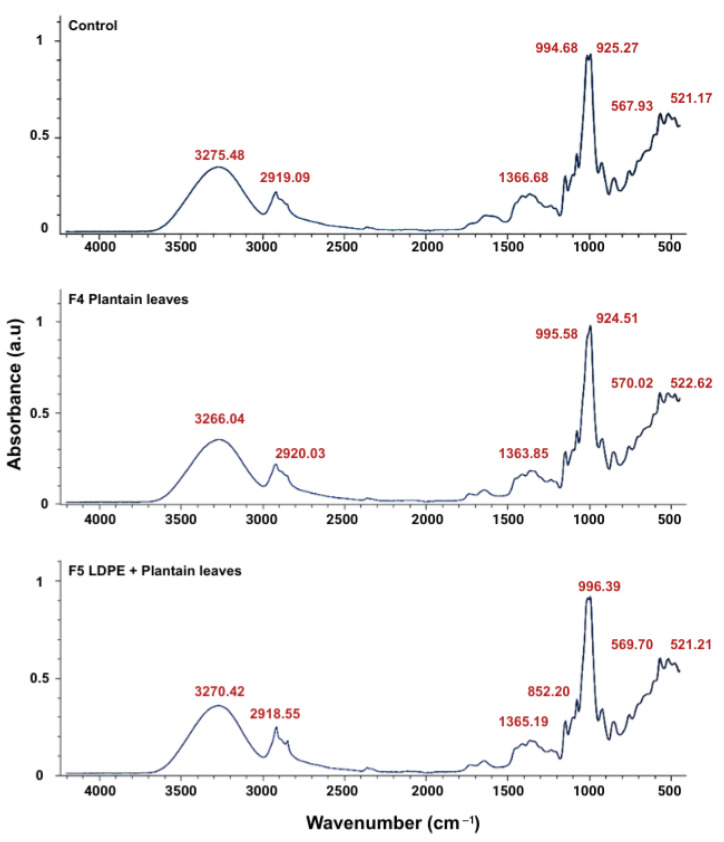
Infrared spectroscopy (absorbance) of compounded TPS films with different formulations: control, ground plantain leaves (F4), and LDPE powder + ground plantain leaves (F5).

The results of the FTIR analysis (Figure 6) confirmed that cassava starch remains the dominant structural component in all films, with a consistent main peak of around 995 cm^−1^, corresponding to the C-O-C stretching of starch [87]. While additives such as beeswax, ground plantain leaves, and LDPE introduce minor variations in specific regions, including C-H (2919 cm^−1^) and O-H (3270–3275 cm^−1^) stretching, these differences remain subtle due to their low concentrations. The film obtained with the F5 formulation exhibited a more pronounced impact in the C-H region, reflecting the polyethylene contribution. At the same time, vibrations in the 500–600 cm^−1^ range indicated interactions between starch chains and additives [88,89]. In any case, the spectra obtained for the two formulations and the control are very similar, which suggests that additives and reinforcing materials did not significantly alter the structure of the starch matrix. This may be of special interest at the time of the film’s biodegradation after use.

#### 3.4.2. Surface and Cross-Sectional Morphology by SEM

**Figure 7:**
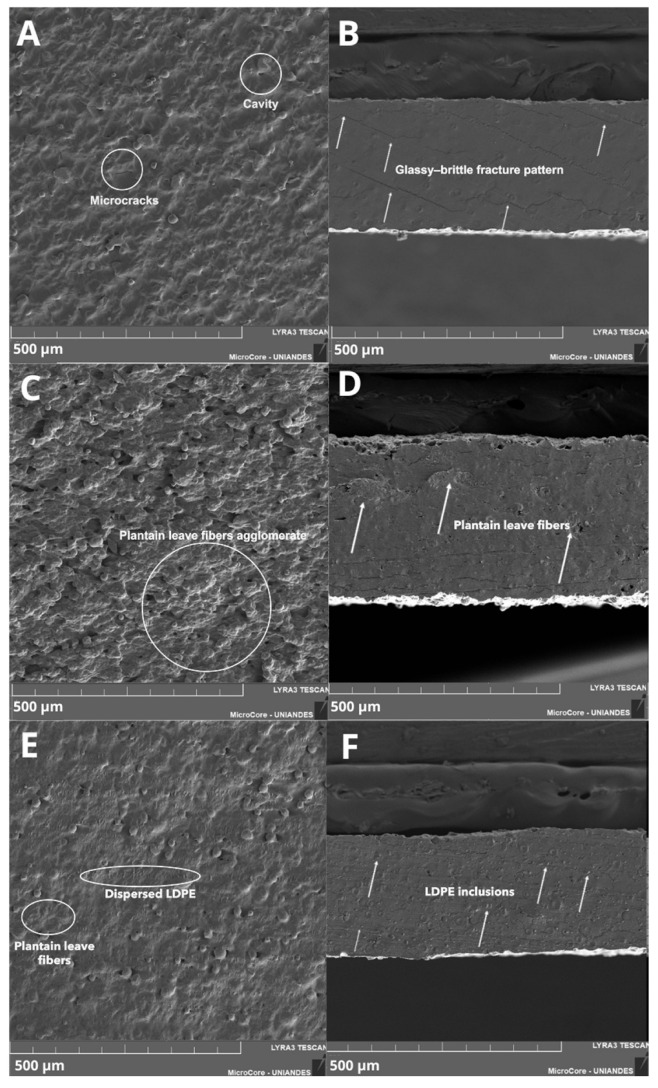
Scanning electron microscopy (SEM) images (300× magnification) (secondary electron mode) of thermoplastic starch (TPS) films with different formulations. Control films, surface (**A**) and cross-sectional (**B**); TPS reinforced with powdered plantain leaves and beeswax (F4), surface (**C**) and cross-sectional (**D**); and TPS reinforced with powdered plantain leaves and LDPE (F5), surface (**E**) and cross-sectional (**F**).

SEM analysis of the control film (cassava starch/glycerol TPS) reveals a highly gelatinized, continuous matrix without visible residual granules, confirming adequate starch destructuring during the extrusion process (Figure 7A,B). However, the presence of surface microcracks, localized cavities, and a glassy-brittle fracture pattern suggests a polymer network dominated by hydrogen bond interactions [81], with limited deformation capacity under hygrothermal gradients. Although these microcracks are partly attributable to SEM-induced artifacts, their higher incidence in the control formulation is consistent with the absence of hydrophobic phases that stabilize the matrix against moisture-induced stress. These defects act as preferential pathways for water vapor diffusion and as stress concentrators, explaining the high permeability and rapid mechanical degradation observed under humid conditions [81,90].

In formulation F4 (TPS + plantain leaf fibers + beeswax), SEM micrographs show a transition to a rougher and more heterogeneous architecture, with elongated lignocellulosic structures embedded in the matrix and local evidence of interfacial discontinuities (Figure 7C,D). These features reflect the limited chemical affinity between the hydrophilic starch matrix and the reinforcements, a common phenomenon in biocomposites without specific compatibilization treatments [91,92]. Nevertheless, the absence of severe macroagglomerates and the relatively uniform distribution of hydrophobic inclusions suggest that the inclusion of Span 80 and the extrusion process facilitated an acceptable physical dispersion of the wax. This dispersion increases the tortuosity of the diffusion path, contributing to the observed improvement in mechanical and barrier properties, as reported for TPS reinforced with natural fibers and waxes [93,94]. Such controlled heterogeneity is characteristic of lignocellulosic-reinforced TPS systems processed without chemical compatibilization and does not compromise film continuity.

The F5 formulation (TPS + plantain leaf fibers + LDPE) exhibits the most compact and cohesive morphology, with discrete phases distributed throughout the cross-section (Figure 7E,F), consistent with the hydrophobic inclusions of LDPE dispersed within the starch matrix. Even without chemical compatibilizers, this microstructure suggests a localized reinforcement and barrier mechanism where LDPE inclusions reduce microdefect connectivity, block microcrack propagation, and limit moisture diffusion. This behavior is consistent with previous reports showing that enhanced matrix densification and homogeneous dispersion of reinforcing phases, as observed by SEM, are key factors governing barrier performance in starch-based composites [95,96]. Overall, SEM observations confirm that matrix densification and effective physical dispersion of hydrophobic phases—facilitated by the flat sheet extrusion process—determine the functional improvements observed in F4 and, especially, F5.

#### 3.4.3. Thickness, Color, and Transparency

Film thickness obtained for the control samples and the formulations 4 and 5 are shown in Table 3. The thickness that could be achieved was directly related to the cohesion and tensile strength of each formulation evaluated. In all tests, an attempt was made to gradually accelerate the calendering speed while keeping the film uniform and without breaks. The control film exhibited the most significant thickness due to its lower mechanical strength, which restricted the calendering speed up to 2 ± 0.5 rpm. Incorporating the beeswax and powdered plantain leaves in F4 enhanced mechanical resistance, increasing the calendering speed up to 3 ± 0.5 rpm without a significant thickness reduction compared to the Control. However, adding LDPE microparticles in F5 further improved mechanical properties, enabling a higher calendering speed of 4 ± 0.5 rpm and resulting in a substantial thickness reduction compared to the Control. The F5 formulation showed greater mechanical resistance and greater cohesion in its structure, such that it can withstand greater pulling forces during calendering, obtaining a lower thickness. Minor thickness variability can be attributed to local differences in melt cohesion during film stretching in the flat sheet extrusion and calendering of the reinforced thermoplastic starch samples.

The color analysis of the extruded films reveals differences among the formulations, reflecting the direct influence of additives on the starch matrix. The control sample (without reinforcement materials), with the highest lightness (L* = 39.49), appears whitish-transparent due to the absence of colorant components. F4 exhibits the lowest lightness (L* = 28.25) and the highest values for the chromatic coordinates a* (4.09) and b* (7.91), resulting in a dark brown color intensified by the combination of beeswax and a higher concentration of pulverized plantain leaves, which contribute warm reddish and yellowish tones. In contrast, for the F5 films, a more balanced color was observed, presenting a lighter brown shade (L* = 33.94), a lower red tone (a* = 3.06), and a moderately higher yellow hue (b* = 9.74). This behavior can be attributed to a reduced proportion of plantain leaves and the predominance of beeswax. The F5 formulation resulted in the most homogeneous and uniform color for the film obtained, which corresponds with literature findings that highlight how adjustments in natural additive proportions optimize both color and optical properties in biopolymers [97,98].

Regarding transparency, the analysis of variance (ANOVA) revealed statistically significant differences in the luminous transmittance among treatments (*p* < 0.05) associated with the incorporation of reinforcing agents. The control formulation exhibited the highest transparency, whereas the reinforced formulations (F4 and F5) showed a significant reduction and did not differ statistically from each other according to Tukey’s test. This decrease in transparency is attributed to the physical obstruction of the optical path by lignocellulosic fibers, which increase light scattering due to their dispersed bulk state within the starch matrix [99]. Similar behavior has been reported by Ban et al. [100], who observed that the inclusion of cellulosic reinforcements promotes opacity through enhanced interfacial scattering. Although transparency is reduced, this effect is technically advantageous, as it limits radiation transmission and improves the film’s protective capacity against UV-induced degradation.

#### 3.4.4. Permeability and Adsorption of Water Vapor

The results in Table 3 indicate significant differences in moisture permeation among the films with the different formulations, with the control showing the highest WVTR and Q_H2O_. The sample corresponding to formulation F4 showed a moderate reduction in moisture permeation, which can be attributed to the presence of beeswax and lignocellulosic particles with more hydrophobic characteristics [93,101]. Likewise, F5 film exhibited a significant reduction in permeation, approximately a quarter of the control, which can be explained by the presence of LDPE and its higher hydrophobicity. The results correspond to those reported by Savadekar and Mhaske [102], Malmir et al. [103], and Raj et al. [104] who evaluated the inclusion of reinforcing components such as nano-cellulose fibers, PBHV, and LDPE, reducing water vapor permeation to WVTR values of 15–100 g m^−2^ d^−1^ at 38 °C and 0–90% RH. WVTR showed statistically significant differences among all formulations, reflecting an apparent reduction in water vapor mass transfer with the incorporation of reinforcing components. In contrast, the water vapor permeability coefficient (Q_H2O_) did not differ significantly between F4 and F5 despite their different mean values, as Q_H2O_ is normalized by film thickness. The higher thickness variability of F4 led to overlapping confidence intervals with F5, explaining the shared statistical grouping at the applied confidence level.

For moisture adsorption, significant differences were observed between the formulations and changes over time, specifically for the control and F4 films. The control sample showed the highest water vapor adsorption with values of 0.48 g g^−1^ at 6 h and 0.66 g g^−1^ at 24 h at the test conditions (38 °C and 80% RH). For this film, as moisture adsorption occurred, significant material degradation was also observed, as the film lost its structure and showed signs of cracking, confirming its poor applicability in humid environments due to the high hydrophilicity of the raw starch because of the high presence of hydroxyl groups [105,106]. Film F4 showed lower moisture adsorption on average, 40% of the value obtained with the control film, which can be explained by considering the greater hydrophobicity conferred by the beeswax and lignocellulosic particles as previously mentioned [94,107]. However, less structural degradation was observed in the film after 24 h due to the conditions evaluated. This structural degradation in the case of the control and F4 films can also be related to the increase in the moisture adsorption rate over time, which is related to the increase in the hydrophilicity of the structure as the colloidal dispersion between the starch particles and the reinforcement materials breaks down and loses cohesion due to the adsorbed moisture. On the other hand, sample F5 showed the lowest moisture adsorption (0.1 g g^−1^) and also greater structural stability under the conditions evaluated, with no signs of disintegration and a balance in the adsorption rate after 24 h of testing. In this case, the presence of LDPE resulted in increased resistance to moisture, given its low chemical affinity and solubility [55]. Likewise, the greater stability and lower moisture adsorption in F5 can also be attributed to the lower glycerol content compared to the control and F4, considering the high chemical affinity of glycerol itself for water [27].

#### 3.4.5. Mechanical Properties

**Figure 8:**
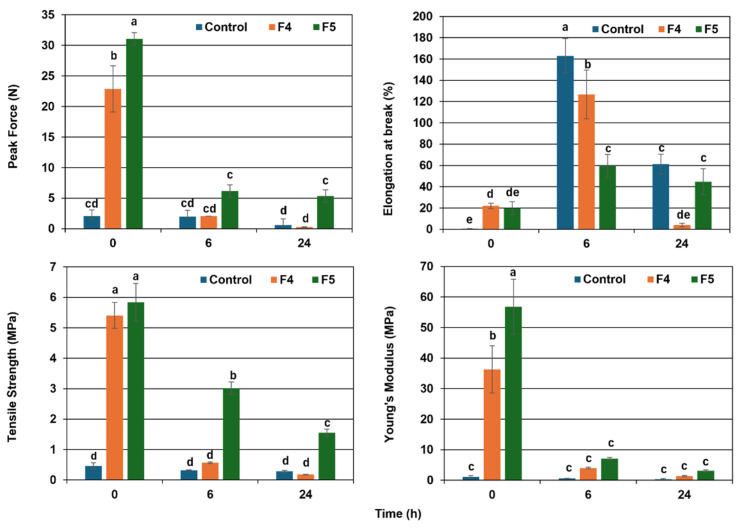
Evolution of mechanical Properties of TPS films with different formulations: control, ground plantain leaves (F4), and LDPE powder + ground plantain leaves (F5) at 35 °C and 75% RH. Each subplot represents an independent mechanical property. Lowercase letters indicate statistically significant differences among formulations at the same time for each property (*p* < 0.05) by Tukey’s HSD test. SD included for *n* = 5.

Mechanical analysis at 35 °C and 75% RH (Figure 8) revealed that the control formulation rapidly lost strength due to high moisture adsorption. Reinforced formulations showed significantly improved stability; F4 provided a balanced profile with higher rigidity than the control and greater flexibility than F5, attributed to the moisture-barrier effects of beeswax and plantain fibers. F5 exhibited the highest initial strength (5.8 MPa tensile strength and 56 MPa Young’s modulus) and superior resistance to degradation over time. This performance, driven by the LDPE reinforcement, aligns with literature indicating that minor additions of synthetic polymers enhance biopolymer moisture resistance [108,109]. In the Amazonian context, using 2% (*w*/*w*) LDPE represents a transitional, application-driven strategy. This approach reduces non-biodegradable content from 100 to 2% while ensuring the mechanical stability required for functional packaging in tropical environments, as supported by previous carbon footprint assessments in the region [110].

Regarding elongation, the control formulation initially exhibited the highest flexibility but quickly lost cohesion, highlighting its fragility under humid conditions. While flexibility is desirable in flexible packaging, excessive elongation can lead to significant deformations under load, compromising functionality. In any case, for the three formulations evaluated in this test, the decline in their mechanical properties was evident and corresponded to the increase in the amount of moisture adsorbed over time (Table 3). This trend, common in moisture-absorbing biopolymers such as cassava starch, reduces structural stiffness, making them less suitable for prolonged stress exposure [111]. Thanks to its hydrophobic components, F4 maintained a balanced performance, with moderate elongation and lower mechanical strength decreasing along the test, which can be attributed to the function of beeswax and the powdered fiber of plantain leaves in providing a more cohesive structure and a greater barrier to moisture adsorption. This increase in strength is more evident for F5 with the inclusion of LDPE in addition to the other reinforcement components already mentioned. This formulation resulted in moderate elongation, preventing excessive flexibility, and a significantly higher Young modulus, indicating greater deformation resistance while maintaining the required flexibility. Likewise, the F5 film retained a higher part of its initial mechanical resistance throughout the test at high humidity and temperature compared to the other formulations, as shown in Figure 8.

All the above correspond with what has been seen during the production of the heat-sealed bags, where the bags corresponding to formulations 4 and 5 contained at least 1 kg of material for 24 h (Figure 5). These results were similar to those obtained by other authors for TPS films reinforced with cellulose nanofibers or LDPE [112,113]. In these studies, tensile strength of 8.3–11.8 MPa and elongation to break of 8.9–55.2% were obtained, achieving an increase in mechanical resistance compared to unreinforced films.

Overall, the results showed that the starch obtained from the different small indigenous Amazonian producers could be used as a mixture to obtain single and reinforced TPS films, confirming the viability of diversifying raw material sources without compromising product quality. Such diversification facilitates large-scale biodegradable packaging production and encourages active local community participation in the supply chain, promoting economic development within cassava-producing regions.

On the other hand, the results showed that TPS formulations that included reinforcing compounds such as beeswax, ground plantain leaf fibers, and LDPE powder led to films with improved barrier and resistance to moisture, lower moisture adsorption, and improved tensile strength compared to unreinforced TPS films. The results showed acceptable performance in heat sealing and load capacity for bags made with TPS formulations with the reinforcements under the climatic conditions typical of humid tropical areas such as the Amazon, as previously described. This is a promising result for these regions, as the reinforced bags could replace single-use flexible packaging with short service lives. The results obtained can be a basis for exploring the inclusion of biodegradable polymers (polybutylene adipate terephthalate—PBAT, Polybutylene succinate—PBS) as an alternative to the powdered LDPE, thus having bags with higher biodegradability/compostability after their final disposal.

## 4. Conclusions

Cassava starch obtained from small indigenous Amazonian plantations can be successfully conditioned and used to make flexible packaging by extrusion. The cassava starch-based formulations analyzed in this study displayed distinct behaviors in terms of mechanical properties, moisture resistance, infrared spectroscopy, and optical characteristics. A direct relationship was observed between the use of reinforcing materials and the functionality and obtainability of the films and flexible pouches by heat sealing.

The control formulation, which contained only TPS without reinforcements, was notable for its structural fragility, high adhesiveness, and susceptibility to moisture, making it unsuitable for practical applications. Using ground plantain leaf particles and beeswax showed improvements in strength and functionality, although their susceptibility to moisture remains significant. Including LDPE powder in small quantities (2% *w*/*w*) with a balanced wax ratio and a lower content of ground plantain leaves presented the best overall performance, combining mechanical stability, greater moisture resistance, uniformity in particle and color distribution, and improved heat sealability. These characteristics demonstrate that this reinforced TPS formulation can be a good alternative for flexible packaging based on biodegradable materials that can be used in the retail trade of products such as food in places like the Amazon with high humidity and temperature environmental conditions.

## Figures and Tables

**Table 1:** Pre-selected formulations evaluated for obtaining reinforced thermoplastic starch films and flexible packages.

Component (% *w*/*w*)	Formulation					
	Control	F1	F2	F3	F4	F5
Cassava starch	66.6	63	63	64.9	60.1	63.9
Water	7.4	7	7	6.9	6.9	7.1
Glycerol	25	25	25	25	25	23
Sunflower oil	0	4	0	0	0	0
Beeswax	0	0	4	2	4	2
Hydrolyzed latex	0	0	0	0.2	0	0
Powdered plantain leaves	0	0	0	0	2.5	1
Powdered LDPE	0	0	0	0	0	2
Citric acid	1	1	1	1	1	1
Span 80	0	0	0	0	0.5	0.5

**Table 2:** Physicochemical characterization of the mixture of raw polyclonal cassava starch obtained from Amazonian indigenous communities.

Parameter (% *w*/*w*)	
Moisture *	44.15 ± 0.27
Carbohydrates **	97.89 ± 0.72
Ash **	0.29 ± 0.02
Ether Extract **	0.38 ± 0.02
Fiber **	0.13 ± 0.00
Protein **	1.31 ± 0.30

Standard deviation (SD) included for *n* = 10, * Moisture content on a total basis; ** Values on a dry basis.

**Table 3:** Thickness, color, transmittance, water vapor transmission rate, and water adsorption of compounded TPS films with different formulations: control, ground plantain leaves (F4), and LDPE powder + ground plantain leaves (F5).

Property	Control	F4	F5
Thickness (mm)	0.32 ± 0.04 a	0.30 ±0.02 a	0.26 ± 0.02 a
Lightness (L*)	39.49± 0.91 a	28.25 ± 0.21 b	33.94± 0.73 c
Chromatic coordinate a*	0.24 ± 0.04 a	4.09 ± 0.22 b	3.06 ± 0.02 c
Chromatic coordinate b*	2.12 ± 0.16 a	7.91 ± 0.68 b	9.74 ± 0.37 c
Transmittance (%)	60.24 ± 0.99 a	67.94 ± 0.68 b	78.82 ± 0.44 b
WVTR (g m^−2^ d^−1^)	1487 ± 287 a	716 ± 184 b	367 ± 79 c
Q_H2O_ (g mm m^−2^ atm^−1^ d^−1^)	24,845 ± 7980 a	13,366 ± 4585 b	5095 ± 1499 b
Water adsorption (g g^−1^)			
6 h	0.48 ± 0.01 Aa	0.17 ± 0.02 Ab	0.10 ± 0.02 Ac
24 h	0.66 ± 0.01 Ba	0.30 ± 0.01 Bb	0.11 ± 0.02 Ac

WVTR, Q_H2O_, and water adsorption were measured at 35 °C and 75% RH. Different lowercase letters in the same row indicate significant differences (*p* < 0.05) between treatments (*p* < 0.05) by Tukey’s HSD test. For the moisture adsorption, different uppercase letters in the same column indicate significant differences between treatments (*p* < 0.05) for each measurement time. Standard deviation (SD) included for *n* = 3.

## Data Availability

The original contributions presented in this study are included in the article. Further inquiries can be directed to the corresponding author.
